# A Multidisciplinary Approach of Type 1 Diabetes: The Intersection of Technology, Immunotherapy, and Personalized Medicine

**DOI:** 10.3390/jcm14072144

**Published:** 2025-03-21

**Authors:** Denisa Batir-Marin, Claudia Simona Ștefan, Monica Boev, Gabriela Gurău, Gabriel Valeriu Popa, Mădălina Nicoleta Matei, Maria Ursu, Aurel Nechita, Nicoleta-Maricica Maftei

**Affiliations:** 1 Department of Pharmaceutical Sciences, Faculty of Medicine and Pharmacy, “Dunărea de Jos” University, 800008 Galati, Romania; denisa.batir@ugal.ro (D.B.-M.); naron@ugal.ro (N.-M.M.); 2 Research Centre in the Medical-Pharmaceutical Field, Faculty of Medicine and Pharmacy, “Dunărea de Jos” University, 800008 Galati, Romania; 3 Department of Morphological and Functional Sciences, Faculty of Medicine, and Pharmacy, “Dunărea de Jos” University, 800008 Galati, Romania; gabriela.gurau@ugal.ro; 4 Clinic Laboratory Department, Clinical Hospital of Children Hospital “Sf. Ioan”, 800487 Galati, Romania; 5 Department of Dental Medicine, Faculty of Medicine and Pharmacy Galați, “Dunărea de Jos” University, 800008 Galati, Romania; gabriel.popa@ugal.ro (G.V.P.); madalina.matei@ugal.ro (M.N.M.); 6 Clinical Medical Department, Faculty of Medicine and Pharmacy, “Dunărea de Jos” University, 800008 Galati, Romania; maria.ursu@ugal.ro (M.U.); aurel.nechita@ugal.ro (A.N.)

**Keywords:** type 1 diabetes, continuous glucose monitoring, insulin therapy, future solutions in T1D therapy

## Abstract

**Background:** Type 1 diabetes (T1D) is a chronic autoimmune disorder characterized by the destruction of pancreatic β-cells, leading to absolute insulin deficiency. Despite advancements in insulin therapy and glucose monitoring, achieving optimal glycemic control remains a challenge. Emerging technologies and novel therapeutic strategies are transforming the landscape of T1D management, offering new opportunities for improved outcomes. **Methods:** This review synthesizes recent advancements in T1D treatment, focusing on innovations in continuous glucose monitoring (CGM), automated insulin delivery systems, smart insulin formulations, telemedicine, and artificial intelligence (AI). Additionally, we explore biomedical approaches such as stem cell therapy, gene editing, immunotherapy, gut microbiota modulation, nanomedicine-based interventions, and trace element-based therapies. **Results:** Advances in digital health, including CGM integration with hybrid closed-loop insulin pumps and AI-driven predictive analytics, have significantly improved real-time glucose management. AI and telemedicine have enhanced personalized diabetes care and patient engagement. Furthermore, regenerative medicine strategies, including β-cell replacement, CRISPR-based gene editing, and immunomodulatory therapies, hold potential for disease modification. Probiotics and microbiome-targeted therapies have demonstrated promising effects in maintaining metabolic homeostasis, while nanomedicine-based trace elements provide additional strategies to regulate insulin sensitivity and oxidative stress. **Conclusions:** The future of T1D management is shifting toward precision medicine and integrated technological solutions. While these advancements present promising therapeutic avenues, challenges such as long-term efficacy, safety, accessibility, and clinical validation must be addressed. A multidisciplinary approach, combining biomedical research, artificial intelligence, and nanotechnology, will be essential to translate these innovations into clinical practice, ultimately improving the quality of life for individuals with T1D.

## 1. Introduction

Diabetes is a metabolic disorder with multiple causes, characterized by chronic hyperglycemia and alterations in glucose, lipid, protein, and electrolyte metabolism due to defects in insulin secretion and/or action. According to the World Health Organization (WHO), prolonged elevated blood glucose levels can cause severe damage to the heart, blood vessels, eyes, kidneys, and nerves [1]. Diabetes is classified into three major types based on its pathophysiology: type 1 diabetes (T1D), type 2 diabetes, and gestational diabetes mellitus [2,3]. Among these, T1D is the most common form in children and adolescents, although it can occur at any age [4,5]. Petersmann et al. highlighted that T1D results from autoimmune-mediated β-cell destruction, leading to absolute insulin deficiency [6]. Recent WHO data indicate a significant rise in T1D incidence, particularly among the pediatric population [7]. Furthermore, Krzewska and Ben-Skowronek reported that T1D is the second most prevalent chronic health condition affecting individuals under 18 years of age [8].

The International Diabetes Federation (IDF) estimated that in 2021, approximately 537 million people worldwide were living with diabetes, with projections reaching 643 million by 2030 and 783 million by 2045 [9]. Moreover, in 2022, there were 8.75 million individuals diagnosed with T1D globally [10]. According to *The Lancet*, the number of adults with diabetes has exceeded 800 million, a fourfold increase since 1990, making diabetes one of the leading causes of mortality worldwide [11].

Recent advancements in T1D treatment are increasingly embracing a multidisciplinary approach that integrates technology, immunotherapy, and personalized medicine [12]. Technology-driven innovations, such as CGM systems, hybrid closed-loop insulin delivery systems, and AI-based glucose prediction models, are significantly enhancing glycemic control and minimizing disease burden for patients [13,14]. These tools enable real-time data collection, individualized insulin dosing, and improved patient adherence, leading to better long-term outcomes [15]. At the same time, immunotherapy is emerging as a potential game-changer in T1D management, aiming to modulate the immune system to preserve or restore pancreatic β-cell function [16]. Novel immunotherapeutic strategies—including monoclonal antibodies, antigen-specific immunotherapy, and regulatory T-cell (Treg) therapy—are currently being explored in clinical trials to slow or halt the progression of β-cell destruction [17]. Additionally, personalized medicine is revolutionizing T1D care by tailoring treatments based on individual genetic, immunological, and metabolic profiles. Advances in gene-editing techniques, such as CRISPR-based β-cell regeneration and gut microbiome-targeted interventions, are paving the way for more patient-specific treatment strategies [18]. Together, these three fields—technology, immunotherapy, and personalized medicine—are driving a paradigm shift in T1D care, moving toward a future where disease management is more effective, individualized, and proactive. However, challenges such as accessibility, cost, and regulatory approval remain critical hurdles that must be addressed to make these advancements widely available.

T1D is a major chronic disease affecting children, with an estimated 651,700 cases among children and adolescents globally in 2021. By 2040, the prevalence is expected to reach 13.5–17.4 million, with the highest increases predicted in low- and middle-income countries [19]. In 2021, approximately 108,300 new cases of T1D were diagnosed among children under 15 years old, a number that rises to 149,500 when extending the age range to individuals under 20 years [20,21]. Ogle et al. noted that T1D incidence is highest among populations of Northern European descent and in several North African and Middle Eastern countries. Additionally, India and the United States report the highest number of annual incident cases among children under 15 years, with 19,194 and 15,288 cases, respectively [20].

A T1D diagnosis at a young age necessitates a significant period of adjustment for both the child and their family. Children and adolescents with T1D often face emotional challenges, particularly when diagnosed early, making parental support essential for disease management [22,23,24]. Studies indicate that adolescents with T1D have higher levels of anxiety [25], increased risk of emotional and behavioral disorders [26], and lower self-esteem and self-image [27], all of which can affect adherence to therapeutic and dietary regimens [28]. Moreover, depression and diabetes frequently coexist, with each condition potentially exacerbating the other [29].

The management of T1D primarily relies on intensive insulin therapy, which includes multiple daily injections or insulin pump therapy. Effective treatment requires ongoing education, adherence to a balanced diet, and regular physical activity to minimize cardiometabolic risks [30,31]. However, adolescence presents unique challenges due to the desire for independence and increased psychological stress, which may lead to risky behaviors such as insulin omission, resulting in complications like diabetic ketoacidosis [32]. Additionally, the rapid endocrinological changes during puberty increase insulin resistance, necessitating frequent insulin adjustments. Complications such as dermatological reactions to therapeutic devices can further impact glycemic control and psychological well-being [33,34]. Prolonged diabetes duration and suboptimal glycemic control can also contribute to early-onset chronic complications, including retinopathy, nephropathy, peripheral neuropathy, and cardiovascular autonomic neuropathy, significantly affecting both patients and caregivers [35,36].

This narrative review aims to provide a comprehensive analysis of the clinical and psychological challenges associated with T1D during adolescence. By synthesizing current evidence, this paper highlights the primary difficulties faced by young individuals with T1D and explores potential strategies to optimize disease management. The review also examines advancements in insulin therapy, technological innovations in diabetes care, and psychosocial interventions that may enhance treatment adherence and quality of life for adolescents with T1D. Through this comprehensive approach, we aim to contribute to the existing body of literature and support healthcare professionals in developing more effective, patient-centered interventions for this vulnerable population.

To conduct this narrative review, we performed a comprehensive literature search focusing on original research articles related to T1D in children and adolescents. Our selection criteria included studies on insulin therapy, insulin pumps, and advanced medical technologies for insulin infusion. We prioritized peer-reviewed publications from the past decade (2014–2025) written in English, without geographic restrictions. The initial search was conducted using Google Scholar, followed by a more refined selection from three major academic databases: PubMed^®^/MEDLINE, ScienceDirect, and Web of Science^®^. The search strategy incorporated the following keywords: *type 1 diabetes technology, continuous glucose monitoring system, automated insulin delivery, continuous insulin infusion, patch insulin pump, hybrid closed-loop insulin pump, closed-loop insulin pump, artificial pancreas, and bionic pancreas.* Only articles published in English were considered, and the final database search was completed on 30 January 2025.

## 2. The Role of Smart Technology in T1D: CGM, Insulin Pumps, and AI Systems

Advancements in diabetes technology have significantly improved disease management and clinical outcomes. The development of automated insulin delivery systems has been a game changer, aiming to enhance metabolic control and quality of life for individuals with T1D. The most effective and reliable therapy for improving HbA1c levels, reducing hypoglycemia, and maintaining glucose within the target range is CSII, commonly known as insulin pump therapy, combined with CGM systems [7]. Additionally, artificial pancreas (AP) systems, also referred to as closed-loop systems (CLS), are now a promising solution for pediatric patients, offering automated and adaptive insulin delivery based on real-time glucose levels. The integration of smartphone-connected diabetes management devices represents a significant step forward in T1D therapy, providing patients and caregivers with real-time data and remote monitoring capabilities.

These advancements, along with personalized insulin regimens and emerging digital tools, are reshaping the landscape of diabetes care, offering greater flexibility, improved adherence, and better long-term health outcomes.

However, despite these promising developments, several challenges remain. CGM and automated insulin delivery systems, while improving glycemic control, still face limitations related to high costs, insurance coverage disparities, and patient adherence. Additionally, accuracy issues in CGM devices, particularly during rapid glucose fluctuations, can lead to dosing errors that impact metabolic control [37]. Similarly, while AI-driven diabetes management provides predictive analytics for glucose trends and insulin adjustments, concerns about data privacy, algorithmic bias, and regulatory hurdles must be addressed before widespread clinical adoption [38]. Nevertheless, ensuring equitable access, addressing technological limitations, and refining regulatory pathways will be essential to maximize their potential impact on clinical practice [39].

Regarding glycemic targets and treatment guidelines, recent recommendations specify optimal glucose targets, such as the following:

- Fasting glucose levels should be maintained between 3.9 and 7.8 mmol/L (70–140 mg/dL) [40].
- For children under 7 years of age, a tighter time interval is recommended, ensuring that at least 50% of CGM readings fall within 3.9–7.8 mmol/L (70–140 mg/dL) or 70% within the range of 3.9–10 mmol/L (70–180 mg/dL) [41].
- ISPAD recommends maintaining HbA1c levels below 53 mmol/mol (<7.0%) to prevent long-term microvascular and macrovascular complications [40].

### 2.1. Continuous Glucose Monitoring (CGM) Systems

Over the past 15 years, advancements in accuracy, accessibility, and insurance coverage have significantly contributed to the widespread adoption of CGM in pediatric diabetes care [42]. Data from the SWEET Registry, which includes centers in Europe, North America, Australia, and the Middle East, confirm a steady increase in CGM and insulin pump usage among children [43]. A population-based study in Australia reported a remarkable rise in CGM uptake among individuals under 21 years old, increasing from 5% to 79%, following the introduction of universal subsidized funding for CGM [44]. However, despite these positive trends, CGM adoption remains inconsistent within the same country, with notable variations between clinical centers and regions [45].

Currently, there are two primary types of CGM available:

- **Professional CGM**: data are recorded over several days and can be accessed only by healthcare providers, typically for retrospective analysis [46].
- **Personal CGM**: Users can view glucose levels in real time, allowing for immediate intervention. This category includes the following:
  ○ Intermittently scanned CGM (IS-CGM) requires manual scanning to retrieve glucose readings [47].
  ○ Real-time CGM (RT-CGM) continuously transmits glucose data to a connected device without scanning [48].

CGM technology can be categorized based on minimally invasive [49] and non-invasive techniques [50]. Several companies have developed minimally invasive CGM systems for diabetes management, as observed in Table 1 [46].

### 2.2. Continuous Subcutaneous Insulin Delivery (CSII) Systems

Continuous subcutaneous insulin infusion (CSII), commonly known as insulin pump therapy, is an advanced method for managing T1D. Compared to multiple daily injections, insulin pumps provide more precise and flexible insulin delivery, helping patients maintain optimal glycemic control [7]. These devices administer insulin in two key ways:

- *Basal insulin infusion*: a continuous supply of small amounts of rapid-acting insulin throughout the day and night to maintain stable glucose levels;
- *Bolus insulin delivery*: a single, larger dose of insulin given at mealtimes or to correct high blood glucose levels [51].

Insulin pumps offer significant advantages, including improved glucose regulation, reduced risk of hypoglycemia, and lower incidence of diabetes-related hospitalizations [52,53]. Research has shown that combining insulin pumps with CGM enhances glycemic outcomes, which has led to the FDA’s approval of integrated CGM systems. These systems can automatically adjust insulin infusion based on real-time glucose readings, suspending or increasing insulin delivery in response to hypoglycemia or hyperglycemia [53]. There are several types of insulin pumps, each offering distinct features and functionalities [52]:

- **Traditional tube pumps**: these pumps feature an insulin reservoir connected to the body via a thin tube, which leads to a cannula (needle or catheter) inserted under the skin.
- **Patch pumps**: These devices are directly attached to the skin and do not require tubing. Insulin is delivered via a short cannula that penetrates the skin.
- **Closed-loop pumps (artificial pancreas systems)**: these fully automated systems combine an insulin pump with a CGM sensor, continuously adjusting insulin doses based on real-time glucose data.
- **Integrated CGM pumps**: while not entirely automated, these pumps work in conjunction with CGM sensors and can suggest insulin dose adjustments based on glucose trends.
- **Mechanical pumps**: these are simpler devices that provide fixed and programmable insulin delivery, often used in specific clinical scenarios [46].

### 2.3. Glucose-Responsive Insulin Delivery System and Artificial Intelligence (AI) in Diabetes Management

Artificial intelligence (AI) is increasingly being explored for its promising applications in data analysis, advanced computing, and predictive modeling. Among the various AI subsets, machine learning and deep learning remain the most widely utilized approaches for enhancing diabetes management. Recent breakthroughs in AI-driven algorithms have significantly improved the prevention of complications, therapeutic strategies, and personalized treatment approaches for T1D [54]. By integrating AI-based solutions into smartphone applications and clinical decision-support systems, patients and healthcare providers can achieve optimized insulin dosing, early detection of glycemic variability, and enhanced self-management strategies [54]. These AI-powered tools can also facilitate early intervention, reducing the risk of hypoglycemia and hyperglycemia-related complications. In addition to glycemic prediction, AI plays a crucial role in precision medicine, particularly through the analysis of genetic and metabolomic data. The increasing availability of digitized glucose monitoring data from T1D patients has fueled AI advancements, enabling the development of personalized treatment algorithms tailored to an individual’s genetic profile, metabolic characteristics, and lifestyle factors. This personalized medicine approach aims to enhance treatment efficacy, minimize adverse effects, and improve overall quality of life [46].

**Figure 1:**
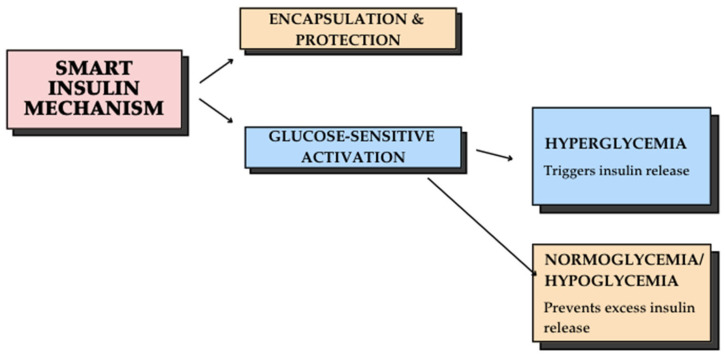
Smart insulin mechanism: a glucose-responsive delivery system.

Smart insulin represents a groundbreaking innovation designed to automatically adjust insulin release in response to blood glucose levels. This technology has the potential to improve glycemic management, reduce hypoglycemia risk, and enhance patient quality of life by minimizing the need for frequent insulin injections and dose adjustments [55]. The core technology behind smart insulin involves the encapsulation of insulin molecules in nanocarriers, which function as glucose-sensitive delivery systems. The process works as follows in Figure 1:

Some of the benefits of smart insulin are reducing the need for frequent injections, improving glycemic control by minimizing hyperglycemia and hypoglycemia risks, and lowering the likelihood of long-term diabetes-related complications [55]. While smart insulin technology holds tremendous potential, challenges remain in optimizing formulation stability, ensuring precise glucose response, and advancing clinical trials for widespread use. Future innovations may integrate biocompatible polymers, AI-driven glucose sensors, and advanced nanotechnology to refine insulin delivery systems. The transition toward an AI-driven framework in diabetes care represents a significant shift in clinical practice, emphasizing individualized treatment protocols and proactive disease management. This is particularly relevant for children and adolescents with T1D, who require adaptive, real-time treatment strategies to account for growth-related metabolic changes and evolving insulin needs. Despite its potential, the implementation of AI in T1D management presents several challenges, including data privacy concerns, algorithmic bias, and the need for large-scale clinical validation. Future research should focus on integrating AI-driven decision-making into routine clinical workflows while ensuring accuracy, reliability, and ethical considerations in diabetes care [54].

### 2.4. Supporting Adolescents in T1D Self-Management: Education and Digital Tools

T1D remains a chronic condition associated with significant morbidity [15], mortality, and reduced quality of life [56,57]. Managing T1D becomes even more complex when it coexists with other medical conditions [58,59,60,61,62]. A good long-term glycemic control can reduce the risk of acute complications (e.g., hypoglycemia, hyperglycemia) and long-term complications (e.g., retinopathy, nephropathy, neuropathy) [63]. Since T1D requires lifelong self-management, frequent consultations with healthcare professionals are essential [64]. Intensive self-care includes continuous blood glucose monitoring, carbohydrate counting, physical activity adjustments, and personalized insulin therapy [65,66].

The transition from childhood to adolescence is a critical phase for individuals with T1D, as they gradually assume greater responsibility for self-management while parents progressively relinquish control [64]. However, this period is often challenging due to the complexity of diabetes management, particularly in the context of suboptimal communication between adolescents and their caregivers. This breakdown in communication can negatively impact health outcomes [67]. Inadequate management during this transition significantly increases the risk of severe hypoglycemia, which may lead to seizures, diabetic ketoacidosis, and comas, potentially resulting in neurological damage or even fatal consequences [68]. Therefore, ensuring effective support during this transition is crucial for safeguarding the adolescent’s health and well-being. Implementing targeted interventions to assist adolescents with T1D in this phase could lead to improved glycemic control, better health outcomes, and enhanced quality of life [69]. Additionally, for young people with diabetes, self-management education can help them gain independence and understand that good diabetes management leads to the prevention of chronic and critical complications and glycemic control [70].

A fundamental aspect of T1D management is self-management education, which should be provided to all children and adolescents diagnosed with the disease [71]. Studies have explored various educational approaches to facilitate self-management in young patients, including personal training, video tutorials [72], mobile phone applications [73], and text messaging interventions [74]. However, findings indicate that while these methods can be beneficial, their effectiveness varies depending on individual learning preferences and engagement levels [71].

The emergence of digital learning tools has paved the way for innovative educational strategies in diabetes management. Among these, digital storytelling has gained attention as a particularly effective method for engaging adolescents. This approach combines narrative elements with multimedia tools, offering a structured way to convey complex medical information in an engaging and accessible manner. Digital storytelling has the potential to enhance knowledge retention and promote behavioral changes, regardless of differences in age, education level, or cognitive abilities [75].

#### 2.4.1. The Role of Digital Storytelling in Diabetes Education

Digital storytelling is a modern educational approach that enables individuals with diabetes to better understand their condition and develop effective self-management strategies. By integrating learning with the enhancement of coping skills, this method serves as a powerful tool to motivate behavioral changes and promote a healthier lifestyle [76]. As a technology-based intervention, digital storytelling combines narrative elements with multimedia capabilities, facilitating the engaging, simplified, and widespread dissemination of educational concepts. This interactive and immersive format encourages active learning, making complex medical information more accessible and relatable [77].

Currently, digital storytelling is widely utilized in adolescent education, with numerous studies examining its effectiveness across various domains. Research highlights its positive impact on academic performance and social intelligence [77], youth achievement [78], and anxiety reduction in patients undergoing cardiac surgery [76] and children with cancer [79]. Additionally, this method has been shown to enhance social skills and cognitive awareness in children and adolescents with autism spectrum disorder [80].

#### 2.4.2. Mobile Technology and IT Innovations in T1D Management

With over 2.7 billion smartphone users worldwide and approximately 0.5 billion individuals already utilizing mobile applications for diet tracking [81], chronic disease management [82,83,84], and physical activity monitoring [85], mobile health (mHealth) technologies have emerged as a promising tool for T1D self-management. In recent decades, advancements in IT have significantly enhanced diabetes care services, offering patients improved access to monitoring tools, personalized interventions, and real-time communication with healthcare providers [86]. Numerous studies have examined the effectiveness of mobile applications and messaging systems as digital health tools for diabetes management, demonstrating their potential to optimize glucose monitoring, treatment adherence, and patient engagement [5,22,23,87,88,89,90,91,92,93,94]. As a result, the availability of T1D-specific applications on platforms such as the iOS App Store and Google Play has expanded significantly [5,23]. These applications—commonly categorized as mobile health (mHealth) apps—are designed to enhance patient education, facilitate communication with healthcare professionals, and promote peer support in an interactive and user-friendly manner [88,95]. This technology is particularly well-suited for children and adolescents, an age group that readily embraces digital tools for daily health management [96].

Table 2 provides an overview of different smartphone applications developed to assist T1D self-management among young individuals.

Auxiliary-style mobile applications represent a promising strategy for improving glycemic control in young patients with T1D, given the widespread use of smartphones in daily life. These apps leverage technology to support self-management, education, and adherence to treatment, offering a convenient and interactive approach to diabetes care.

Den Akker et al. introduced PERMAGON, an integrated gaming and coaching platform designed specifically for adolescents with T1D. The system combines monitoring, educational games, and motivational feedback to enhance self-management skills in both patients and their caregivers [106]. Similarly, Tidepool, a device-agnostic cloud platform, was developed by Neinstein et al. to centralize and analyze data from various diabetic devices. This innovation addresses two major challenges in T1D management: limited access to device-generated data and poor interoperability across different devices [107].

Another notable digital tool is DigiBete, a free self-management and educational application designed for children, adolescents, families (CYPFs), and healthcare professionals (HCPs). Developed in collaboration with both CYPFs and HCPs, this platform provides clinically approved and age-appropriate resources to aid in T1D management. It offers over 200 educational videos covering topics such as carbohydrate counting, sports, and exercise. Additionally, DigiBete allows users to store insulin ratios, dosage information, and pump settings, while also facilitating direct communication between patients and their diabetes care team. A key feature of this application is its multilingual accessibility, supporting British Sign Language, Bengali, Chinese, Arabic, Somali, Tamil, Polish, and Urdu, with further language expansions in progress [5].

Beyond mobile applications, text messaging services have also emerged as effective tools for diabetes education and management. Bin-Abbas et al. implemented a mobile phone messaging service for children and adolescents with T1D, where parents received three types of messages: informational, interactive, and multimedia-based [108]. The study revealed significant benefits, including enhanced communication between patients and healthcare providers during clinic visits as well as significant reductions in HbA1c levels, indicating improved glycemic control and increased parental knowledge of diabetes, leading to better disease management at home [109]. When integrated into mobile applications and patient portals, digital health interventions have proven to be highly effective in enhancing self-efficacy, promoting treatment adherence, and ultimately improving glycemic outcomes [110,111,112]. As the field of digital diabetes care continues to evolve, the incorporation of advanced IT tools and communication platforms holds great potential in optimizing the management of T1D, particularly among adolescents.

#### 2.4.3. The Role of Telemedicine in T1D Management

The COVID-19 pandemic accelerated the adoption of telemedicine, transforming it into a primary mode of diabetes care worldwide [113,114,115]. During lockdowns, diabetes management was conducted almost entirely through virtual consultations, and both patients and healthcare providers became increasingly comfortable with this remote clinical environment [116]. Telemedicine has demonstrated significant benefits in T1D management, particularly in improving insulin adherence (patients receive real-time guidance on insulin dosing adjustments), sick day management (immediate access to medical advice prevents severe complications), and the reduction in unnecessary hospital visits (virtual care decreases emergency admissions). The positive impact of telemedicine lies in the frequent touchpoints between patients and healthcare teams, facilitated through emails, text messaging, and videoconferencing [117,118]. A recent study on children with T1D found that families perceived telehealth as beneficial, citing cost savings, increased flexibility, and more frequent insulin adjustments as major advantages [119]. This shift toward remote monitoring and intervention has led to a surge in telemedicine-focused research, with numerous studies reporting remarkable success in the remote follow-up of pediatric T1D patients during the pandemic [114,120,121]. Several limitations have been identified that may impact the quality of T1D care:

- Lack of physical examination: remote consultations limit physicians’ ability to conduct comprehensive physical assessments, which are critical for detecting diabetes-related complications [122,123].
- Reduced patient-physician interaction: personal contact is a key element in establishing trust and improving patient adherence, which may be compromised in virtual care settings [124].
- Technological barriers: some patients and healthcare providers struggle with limited digital literacy, affecting their ability to effectively utilize telemedicine platforms [123].

While telemedicine continues to evolve as an integral component of diabetes management, future advancements should focus on hybrid care models that integrate in-person consultations with digital health tools, ensuring comprehensive and personalized care for individuals with T1D. Future research should focus on integrating telemedicine with mobile applications and digital tools to enhance comprehensive diabetes care.

### 2.5. Expanding Diabetes Technology Worldwide: Challenges and Opportunities

The integration of AI-driven decision-support systems, CGM, and hybrid closed-loop insulin delivery has significantly advanced T1D management, enhancing glycemic control and reducing the burden of self-care [125]. These technologies enable more precise insulin dosing, leading to fewer hypoglycemic events and improved time-in-range outcomes. However, while their clinical efficacy is well-documented, the successful implementation of these innovations in real-world practice remains contingent upon several factors, including patient adherence, healthcare provider training, and long-term safety validation [126].

Despite their advantages, the high cost and limited insurance coverage continue to pose significant challenges to widespread adoption. This issue is particularly evident in resource-limited settings, where conventional insulin therapy remains the primary treatment option due to financial and accessibility constraints. The availability of specialized diabetes care infrastructure is another major barrier, as advanced diabetes technologies require specific expertise for effective implementation [125]. However, a significant number of healthcare professionals, particularly in low- and middle-income countries, have not received formal training in CGM interpretation, insulin pump therapy, and AI-driven diabetes management [127]. This knowledge gap further limits the accessibility and scalability of these advanced technologies in clinical practice. In addition, data privacy concerns and digital literacy gaps remain significant barriers. Many AI-driven diabetes management tools rely on cloud-based platforms, necessitating secure data-sharing frameworks [128]. However, in regions with weaker cybersecurity infrastructure, compliance with data protection regulations such as GDPR and HIPAA presents additional challenges to adoption [129].

To ensure equitable access to these emerging diabetes technologies, governments and healthcare systems must prioritize cost-reduction strategies, including subsidies for CGM devices and insulin pumps and the expansion of insurance coverage for digital health solutions [130]. Furthermore, regulatory agencies should expedite approval pathways for AI-powered diabetes management tools while maintaining rigorous safety and efficacy standards. Collaborative efforts between healthcare providers, policymakers, and technology developers are essential to streamline these processes and ensure that innovative solutions reach those who need them most [46]. In addition, the adoption of hybrid healthcare models—which integrate telemedicine with in-person diabetes care—may facilitate broader access to specialized diabetes management services, particularly in underserved regions [122]. Digital health literacy programs should also be developed to enhance the ability of both patients and healthcare professionals to effectively utilize emerging diabetes technologies, thereby maximizing their clinical impact and improving long-term health outcomes for individuals with T1D [130].

## 3. Future Solutions in the Therapy of T1D

Managing T1D in children and adolescents presents significant challenges, as it requires achieving optimal glucose control while minimizing hypoglycemia risks. Over the past two decades, diabetes technology has evolved considerably, providing advanced solutions to support glycemic management [131]. The continuous development of diabetes management technologies has led to updates in clinical recommendations, particularly for young patients, as outlined in the International Society for Pediatric and Adolescent Diabetes (ISPAD) Guidelines [30]. These guidelines establish specific glucose targets to improve outcomes and reduce long-term complications.

**Figure 2:**
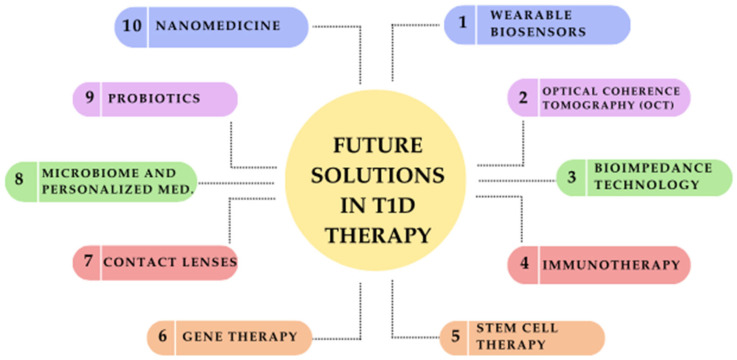
Future solutions in type 1 diabetes (T1D) therapy: a schematic overview of emerging innovations.

Emerging therapies not only improve the quality of life for individuals with T1D but also provide hope for a future where disease management becomes more efficient and less burdensome [132]. Figure 2 represents a schematic diagram of future solutions for T1D.

The landscape of T1D treatment is rapidly evolving, with several innovations ranging from clinically available treatments to experimental therapies still in early research. In Table 3, we categorize advancements into two groups: **emerging therapies** (undergoing clinical trials with potential for near-future implementation) and **experimental therapies** (in early-stage research or preclinical development, requiring further validation before clinical application).

### 3.1. Wearable Biosensors: Non-Invasive Continuous Monitoring

Wearable biosensors are a promising innovation in T1D management, offering a non-invasive and continuous method for monitoring glucose levels. These sensors are designed to detect glucose variations in interstitial fluid by using advanced nanomaterials or chemical biosensors that react to glucose by changing color or emitting light. This direct visual feedback provides real-time insights into glycemic fluctuations [138,139].

Wearable biosensors are typically wirelessly connected to mobile devices, enabling the following:

- Real-time glucose tracking via smartphone applications;
- Automated alerts when glucose levels exceed pre-set thresholds;
- Data storage and analysis to enhance long-term glycemic management.

Compared to traditional glucometers, wearable biosensors eliminate the need for frequent finger pricks, offering a more convenient and user-friendly alternative [140].

### 3.2. Optical Coherence Tomography (OCT) for Glucose Monitoring

Optical coherence tomography (OCT) is an imaging technology that uses light waves to generate high-resolution cross-sectional images of biological tissues. Recent studies have explored its potential for non-invasive CGM, leveraging its high signal-to-noise ratio and superior resolution [21,141].

The advantages of OCT-based glucose monitoring include the following:

- **Completely non-invasive**, eliminating the need for needles or skin punctures;
- **High imaging precision** enables real-time tracking of glucose fluctuations;
- **Enhanced patient comfort** is particularly beneficial for pediatric patients [142].

However, challenges remain, including motion artifacts, temperature sensitivity, and tissue property variations, which affect measurement accuracy. Further technological refinements are needed before OCT-based CGM can be widely implemented [142].

### 3.3. Bioimpedance-Based Glucose Monitoring

Bioimpedance technology measures the electrical resistance of biological tissues when an electric current passes through them. Since bioimpedance variations correlate with glucose fluctuations, researchers have explored its potential for accurate, real-time glucose monitoring [143,144].

Key findings suggest the following:

- Bioimpedance offers a stable and precise estimation of glucose levels, particularly within an optimal frequency range below 40 kHz [145].
- CGM devices based on bioimpedance include sensors, analytical algorithms, and measurement circuits, offering a potential alternative to traditional CGM systems [138].

### 3.4. Immunotherapy: Modulating the Immune Response in T1D

**Figure 3:**
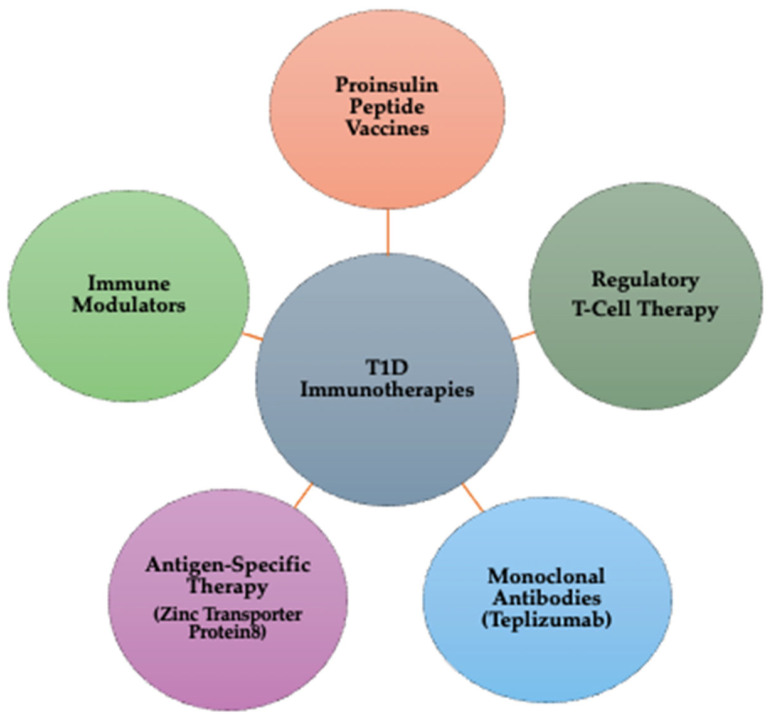
Types of immunotherapies for T1D: mechanisms and targets.

Immunotherapy represents a rapidly advancing field in T1D research, aiming to modify the immune response and prevent the destruction of insulin-producing pancreatic beta cells [146]. T1D is an autoimmune disorder in which the immune system mistakenly targets beta cells, leading to insulin deficiency and lifelong dependence on external insulin administration. Immunotherapy has emerged as a potential solution to slow disease progression or even halt autoimmune destruction [17]. In Figure 3, there is a schematic representation of different types of immunotherapies in T1D.

*The types of Immunotherapies in T1D are outlined below:*

- **Proinsulin peptide (PPI) vaccines:** Proinsulin peptide vaccines train the immune system to tolerate beta cells instead of attacking them. By exposing the immune system to proinsulin fragments, these vaccines help induce immune tolerance, potentially preventing or delaying T1D onset. *Clinical stage:* phase I/II trials are ongoing to assess efficacy and safety [17].
- **Regulatory T-cell (Treg) therapy:** Tregs are a specialized subset of immune cells responsible for suppressing autoimmune reactions. In Treg therapy, patient-derived Tregs are extracted, expanded in a laboratory, and reinfused to restore immune balance. This approach reduces beta cell destruction and modulates immune responses, helping preserve endogenous insulin production. *Clinical stage:* multiple Phase I/II clinical trials are investigating Treg therapy in newly diagnosed T1D patients [147].
- **Monoclonal antibodies (teplizumab therapy):** Teplizumab is a monoclonal antibody therapy that targets CD3 on T-cells, effectively by blocking key immune pathways involved in the attack on beta cells, delaying disease progression in newly diagnosed individuals, and reducing inflammation and modulating the immune system’s response to beta cells. *Clinical stage:* Teplizumab has received FDA approval for delaying the onset of T1D in at-risk individuals. Ongoing studies continue evaluating long-term efficacy [16,148].
- **Antigen-specific therapy— Zinc Transporter Protein 8 (ZnT8) targeting:** ZnT8 is an autoantigen present in prediabetic and diabetic patients, making it a critical target for immunotherapy. ZnT8-based treatments help regulate immune responses, potentially protecting beta cells from further autoimmune damage. Early diagnosis strategies using ZnT8 autoantibodies can also improve predictive screening for T1D risk. *Clinical stage:* preclinical and early-stage Phase I trials [149,150];
- **Immune modulators:** Immune modulation aims to modify the immune response to maintain beta cell function and prevent or delay the onset of the disease. Abatacept, a T-cell co-stimulation blocker, reduces inflammation and lowers the likelihood of autoimmune destruction. Other immunomodulatory drugs are currently being tested to improve beta cell survival and enhance long-term glycemic control. *Clinical stage:* phase II/III trials are evaluating the efficacy in delaying T1D progression [151].

### 3.5. Stem Cell Therapy

Stem cell therapy represents a groundbreaking approach in T1D treatment, aiming to regenerate insulin-producing beta cells in the pancreas. This method holds great potential for restoring endogenous insulin production and improving long-term glycemic control. Current research focuses on utilizing different types of stem cells to replace damaged beta cells and enhance pancreatic function [152]. The types of stem cells used in T1D therapy are the following:

- **Embryonic stem cells (ESCs)** are pluripotent cells capable of differentiating into any cell type, including beta cells. These cells have shown great potential for beta cell regeneration but raise ethical concerns and a risk of immune rejection [153];
- **Induced pluripotent stem cells (iPSCs)** are reprogrammed adult cells that mimic ESCs, offering a patient-specific approach. These cells are generated from the patient’s own cells, reducing the risk of immune system rejection and provide an ethically viable alternative to embryonic stem cells [154];
- **Mesenchymal stem cells (MSCs)** are found in bone marrow, adipose tissue, and umbilical cord blood. These cells exhibit anti-inflammatory and immunomodulatory properties, making them valuable in autoimmune diseases like T1D. Also, they differentiate into multiple cell types, including insulin-producing beta cells [155,156].

Despite promising results in preclinical and clinical trials, challenges remain in ensuring long-term beta cell survival, optimizing differentiation protocols, and addressing immune rejection issues. Future advancements in gene editing (e.g., CRISPR) and tissue engineering could further enhance the success of stem cell-based T1D therapies [18].

### 3.6. Gene Therapy

Gene therapy for T1D is a cutting-edge approach aimed at correcting genetic defects or modifying gene expression to restore insulin-producing beta cell function in the pancreas. This innovative strategy involves introducing, removing, or altering genetic material within a patient’s cells, with the ultimate goal of treating or potentially curing the disease [135]. Key mechanisms of gene therapy in T1D include the following:

- **Gene transfer for beta-cell regeneration** uses viral vectors (e.g., adenoviruses, lentiviruses) to introduce essential transcription factors like PDX1 and MAFA. These factors play a critical role in beta cell development and regeneration, restoring insulin production in individuals with T1D [135].
- **CRISPR-Cas9 gene editing** is a revolutionary gene-editing technique that enables precise genetic modifications. This gene can correct mutations in the insulin gene to ensure proper insulin production. Also, it allows the introduction of protective sequences to shield beta cells from autoimmune destruction [157].
- **Gene therapy for localized immunosuppression** delivers therapeutic genes that produce immunosuppressive proteins (e.g., IL-10 and TGF-beta). Also, it protects beta cells from autoimmune attacks without compromising the body’s immune defense against infections [157].
- **Alpha-to-beta cell conversion** introduces specific genes like ARX and PDX1 to reprogram glucagon-producing alpha cells into insulin-producing beta cells. This technique offers an alternative source of functional beta cells, restoring natural insulin production [18].

Although gene therapy holds immense potential in revolutionizing T1D treatment, several challenges remain: ensuring long-term beta cell survival post treatment, minimizing immune rejection of newly regenerated beta cells, and enhancing gene delivery efficiency to improve treatment outcomes. Future advancements integrating CRISPR technology, stem cell therapy, and improved gene delivery systems could bring gene therapy closer to clinical application as a viable cure for T1D [18].

### 3.7. Contact Lenses for CGM

Contact lenses represent an innovative technology in the management of T1D, offering a non-invasive and continuous method for monitoring blood glucose levels. These smart lenses are designed to detect glucose concentration in tears, providing real-time data that help patients achieve better glycemic control and prevent complications associated with blood glucose fluctuations [158]. The technology behind these lenses relies on microscopic sensors embedded in the contact lens material. These sensors use electrochemical or optical detection systems to measure glucose levels in the tear film. The collected data are then wirelessly transmitted to an external device, such as a smartphone or smartwatch, enabling CGM. When glucose levels deviate from the normal range, the system sends immediate alerts, allowing patients to take timely action [158]. This emerging technology has the potential to revolutionize diabetes self-management by eliminating the need for frequent finger-prick blood tests, making glucose monitoring more convenient and user-friendly. However, challenges such as sensor accuracy, durability, and user comfort must be addressed before these lenses can be widely implemented in clinical practice.

### 3.8. Microbiome and Personalized Medicine

Bioinformatics is an interdisciplinary field that applies computer science and statistical methods to the study of biological data. It plays a crucial role in processing and analyzing vast amounts of biological information, including genome sequencing, proteomics, and transcriptomics data [159]. By utilizing computational tools, bioinformatics enables researchers to efficiently process large-scale datasets, extract hidden patterns, and uncover significant biological insights.

The gut microbiota represents a complex microbial ecosystem composed of bacteria, fungi, viruses, and other microorganisms residing in the human intestine. These microbes play a key role in host metabolism and immune regulation, significantly influencing the development of diabetes and insulin resistance [160]. Disruptions in gut microbiota composition, also known as dysbiosis, have been linked to altered glucose metabolism, inflammation, and insulin resistance, highlighting its importance in metabolic diseases [161].

Bioinformatics analysis offers several advantages in the study of gut microbiota and its association with diabetes and insulin resistance. It enables efficient processing of big data, facilitates multi-level integration of complex datasets, assists in the discovery of biomarkers and therapeutic targets, and enhances predictive modeling and simulation for metabolic disorders [161]. Additionally, bioinformatics tools promote data sharing and collaboration, providing researchers with powerful computational frameworks to decode the intricate relationships between microbiota diversity and metabolic diseases [162].

Since scientific evidence suggests a strong correlation between gut microbiota composition, diabetes, and insulin resistance, bioinformatics has emerged as a cutting-edge approach for gaining deeper insights into the mechanisms by which intestinal microbes influence glucose metabolism. Moreover, personalized medicine is increasingly leveraging microbiome research to develop tailored therapeutic strategies, allowing for individualized interventions based on a patient’s unique microbial composition and metabolic profile [163]. Continued research in this field may pave the way for microbiota-targeted therapies, offering new perspectives for precision medicine in diabetes management [164].

In the literature, there are studies regarding the changes in the diversity and relative abundance of gut microbes that occur in T1D. Factors that could influence the microbiome data and its implications in T1D include data related to participants—age, sex, geographical location, dietary pattern, physical activity, and disease severity—methods used for sample collection, DNA isolation methods, and microbial profiling methods (metagenomics and metabolomics), but also bioinformatics tools used for analysis. Therefore, in future studies, these factors should be taken into account [165].

### 3.9. The Role of Probiotics in T1D Management

Probiotics have emerged as a potential therapeutic approach to restore microbiota balance and support glucose homeostasis [166]. Table 4 provides a structured overview of the mechanisms linking gut microbiota, probiotics, and T1D management, illustrating how microbial interventions may influence diabetes progression and therapy.

Although T1D has been recognized for a long time, the specific mechanisms underlying pancreatic β-cell destruction remain incompletely understood. The most widely accepted explanation is the interaction between genetic susceptibility and environmental triggers, such as viral infections, which can initiate an autoimmune response against pancreatic β-cells [19]. Recent research has established a strong correlation between gut microbiota imbalance (dysbiosis) and T1D development [166]. Compared to healthy individuals, patients with β-cell autoimmunity exhibit a lower abundance of beneficial bacteria (e.g., *Bifidobacterium* and *Lactobacillus*) alongside a notable increase in pro-inflammatory bacteria such as Clostridium and Bacteroides. These microbial changes contribute to intestinal permeability, chronic inflammation, and immune dysregulation, factors known to play a role in T1D progression [35].

Moreover, studies have shown that children with T1D tend to have a reduced diversity of gut microbiota and lower levels of bacteria crucial for intestinal mucosal integrity. The resulting compromised intestinal epithelial barrier leads to increased permeability, which further exacerbates inflammation. This inflammatory state triggers the release of lipopolysaccharides into circulation, a process associated with higher insulin resistance and impaired glycemic control [164,172]. According to Homayouni-Rad et al., emerging evidence suggests that the use of probiotics, prebiotics, or symbiotic may help modulate gut microbiota composition in individuals with diabetes [162]. The administration of specific probiotic strains has been shown to increase the production of short-chain fatty acids (SCFAs), such as butyrate, which plays a key role in maintaining intestinal homeostasis. SCFAs exert their beneficial effects by activating free fatty acid receptors 2 and 3 (FFAR2/3), mechanisms that regulate immune function and autoimmune disease progression, including T1D [173].

Additionally, SCFA-mediated activation of FFAR2/3 has been linked to enhanced production of glucagon-like peptide-1 (GLP-1) from intestinal L cells. Since GLP-1 stimulates insulin secretion from pancreatic β-cells, this process enhances glucose regulation and insulin sensitivity, an effect commonly referred to as the “incretin effect” [174,175,176]. These findings underscore the therapeutic potential of probiotics in preventing or managing T1D by preserving gut microbiota balance and modulating immune responses. However, further clinical trials are needed to establish standardized probiotic formulations and determine their long-term efficacy in diabetes management.

### 3.10. Nanomedicines Based on Trace Elements in Diabetes Management

Trace elements, also known as microelements, are essential chemical substances present in the human body in small amounts, playing a crucial role in growth, development, and metabolic functions. In recent years, nanoparticles derived from trace elements have gained increasing attention as nanomedicines in antidiabetic therapies due to their ability to modulate glucose metabolism through multiple pathways [137].

The mechanisms of action of trace element nanoparticles in diabetes management are outlined below:

- **Blood glucose regulation**: trace element nanoparticles help lower blood glucose levels, reducing hyperglycemia.
- **Improved insulin sensitivity**: these nanoparticles enhance cellular responsiveness to insulin, allowing for better glucose uptake and utilization.
- **Enhanced insulin secretion**: certain trace element-based nanomedicines stimulate pancreatic β-cells to produce and release insulin more efficiently.
- **Alleviation of glucose intolerance**: by modulating glucose absorption and utilization, these nanoparticles help prevent postprandial glucose spikes.
- **Lipid profile improvement**: studies have shown that trace element nanoparticles can positively influence lipid metabolism, reducing the risk of dyslipidemia in diabetes.
- **Anti-inflammatory and antioxidant properties**: nanoparticles derived from trace elements exhibit anti-inflammatory and antioxidant effects, which are crucial in preventing β-cell damage and mitigating diabetes-related oxidative stress [137].

Due to their broad spectrum of benefits, trace element nanoparticles have significant potential as nanomedicines or dietary supplements for effective diabetes management. However, further clinical research and safety evaluations are required to establish optimal formulations, dosages, and long-term effects in diabetes therapy.

## 4. Conclusions

The management of T1D has significantly evolved due to advances in technology, biomedical research, and personalized medicine. This review underscores the role of emerging therapeutic strategies in improving glycemic control, patient adherence, and quality of life. From CGM systems and automated insulin delivery technologies to nanomedicine-based solutions and gene therapy, the future of diabetes care is shifting toward precision medicine and curative interventions.

Technological advancements, such as artificial pancreas systems, smart insulin formulations, and mobile health applications, have facilitated real-time glucose monitoring and personalized insulin adjustments. AI-based algorithms and machine learning models have further enhanced predictive glucose analytics, allowing for proactive management of blood sugar levels. Additionally, telemedicine platforms have expanded access to diabetes education and remote patient monitoring, particularly benefiting children and adolescents with T1D who require adaptive, real-time treatment strategies. Future perspectives include non-invasive glucose monitoring technologies, advanced biosensors, and AI-driven closed-loop insulin delivery systems, which aim to further optimize metabolic control.

Beyond digital innovations, regenerative medicine and immunotherapies are reshaping the long-term therapeutic landscape of T1D. Stem cell-derived β-cell replacement therapies and CRISPR-based gene editing hold promise for restoring insulin production and modulating autoimmune responses. Next-generation immunotherapies, such as antigen-specific monoclonal antibodies, regulatory T-cell (Treg) therapies, and immune tolerance induction strategies, aim to halt disease progression and promote sustained remission. Future research will focus on combining immunotherapy with regenerative approaches, potentially leading to curative solutions for T1D. Additionally, gut microbiota-targeted therapies, including probiotics, prebiotics, and microbiome-based interventions, have also demonstrated potential in reducing inflammation and improving metabolic homeostasis. Nanomedicine-based solutions, such as trace element nanoparticles for insulin delivery and immune modulation, may provide non-invasive, highly effective therapies for diabetes management. Advancements in gene therapy, epigenetic editing, and synthetic biology could open new possibilities for reprogramming immune responses and regenerating pancreatic function.

Despite these scientific breakthroughs, several challenges remain. The long-term efficacy and safety of gene therapies, nanomedicines, and β-cell transplantation require further clinical validation. Additionally, barriers to accessibility, including cost, healthcare infrastructure, and patient adherence, must be addressed to ensure widespread adoption of advanced diabetes treatments. Moving forward, a multidisciplinary and patient-centered approach will be essential in integrating biomedical engineering, artificial intelligence, regenerative medicine, and nanotechnology into clinical practice. Collaborations between researchers, clinicians, biotechnologists, and regulatory agencies will be critical in translating emerging therapies from clinical trials to widespread clinical implementation. By embracing technology-driven solutions, precision medicine, and immune-based interventions, the future of T1D care is moving toward disease modification, prevention, and ultimately, a cure.

## Figures and Tables

**Table 1:** Global manufacturers of CGM devices.

Company Name	Country	Example of CGM Device	Functional Principle	Clinical Relevance and Mechanism
**F. Hoffmann-La Roche**	Switzerland	Accu-Chek CGM System	Utilizes a subcutaneous sensor to measure glucose in interstitial fluid, transmitting data wirelessly to a receiver or smartphone.	Provides real-time glucose tracking and alerts users to prevent hypo- or hyperglycemia.
**A. Menarini Diagnostics**	Italy	GlucoMen Day CGM	Employs a minimally invasive continuous glucose sensor that connects via Bluetooth for real-time monitoring.	Enhances glucose control by reducing the frequency of blood glucose finger sticks.
**B. Braun Melsungen AG**	Germany	Omnitest CGM	Uses an enzymatic biosensor to detect glucose levels in the interstitial fluid, providing continuous data.	Supports proactive insulin adjustments by offering trend insights.
**Echo Therapeutics, Inc.**	USA	Symphony CGM	Implements a transdermal biosensor for non-invasive glucose measurement.	Aims to eliminate discomfort associated with invasive CGM devices.
**Johnson & Johnson**	USA	Animas Vibe CGM	Integrates a real-time glucose sensor with an insulin pump for automated insulin adjustments.	Improves glycemic control through a semi-automated insulin delivery system.
**Medtronic plc**	USA	Guardian Connect CGM	Uses a thin subcutaneous sensor to provide continuous glucose readings to a linked device.	Provides predictive alerts for hypo- and hyperglycemia, improving glycemic stability.
**GlySens Incorporated**	USA	GlySens ICGM System	Features a fully implantable CGM that transmits glucose data continuously.	Reduces the need for sensor replacements, allowing long-term glucose monitoring.
**Senseonics Holdings, Inc.**	USA	Eversense Implantable CGM	Utilizes an implantable fluorescence-based glucose sensor that communicates with a wearable transmitter.	Offers extended wear time (up to 6 months) for reduced device maintenance.
**Abbott Laboratories**	USA	FreeStyle Libre	Uses a filament sensor placed under the skin that is scanned manually for glucose readings.	Eliminates routine fingerstick testing while providing retrospective glucose data.
**LifeScan IP Holdings LLC**	USA	OneTouch CGM System	Employs a subcutaneous electrochemical sensor that transmits glucose levels to a connected app.	Facilitates real-time glucose monitoring with digital tracking for better self-management.
**Terumo Corporation**	Japan	Terumo CGM	Implements an enzymatic reaction-based sensor for continuous glucose tracking.	Ensures high accuracy in glucose detection, aiding in insulin dosing decisions.

**Table 2:** Different smartphone apps for T1D management.

Application Name	Key Benefits	References
**Bant app**	Enhances blood glucose monitoring and self-management.	[5,97]
**Webdia app**	Reduces HbA1c levels in children.	[87]
**MyT1DHero app**	Improves communication between parents and adolescents; enhances adherence to self-care.	[23,98]
**Ana Alsukary app created** **in Saudi Arabia**	Helps children understand their diabetes and adjust their lifestyle.	[24]
**Young with Diabetes app** **(YWD)**	Reduces feelings of loneliness; enhances T1D knowledge and self-management skills.	[88,92,99]
**Diapplo app**	Partially meets children’s educational needs.	[100]
**Mobile Diabetes Advice for Dads (mDAD)**	Assists fathers in understanding and supporting diabetes management.	[89]
**SweetGoals app**	Supports young adults in complying with their medical regimen.	[101]
**Mobile Diab**	Aids in T1D treatment for adolescents.	[86,102,103]
**CanDIT (Canadian Diabetes Incentives and Technology) app**	Highly accepted tool for providing diabetes-related information.	[103]
**iSpy app and glycemic control tools**	Encourages glucose monitoring, assists with data collection, and supports healthy nutrition and medication dosing.	[104,105]

**Table 3:** T1D therapy categorization.

Category	Therapy	Development Stage	Key Benefits	Challenges/Risks	Reference
**Emerging Therapies (In Clinical Trials, Awaiting Approval)**	Beta Cell Regeneration Therapies (Stem Cell-Derived β-Cells)	Phase 1/2 clinical trials	Potential to restore natural insulin production	Risk of immune rejection, ethical concerns	[133]
	Advanced Artificial Pancreas (Next-Gen Hybrid Closed-Loop Systems)	Late-stage development	AI-driven glucose prediction, dual hormone systems	Requires extensive validation, high costs	[125]
	Combination Immunotherapy Approaches	Phase 1/2 trials	Aims to protect β-cells from autoimmune destruction	Long-term effects and patient selection still uncertain	[134]
**Experimental Therapies (Preclinical or Early Research)**	CRISPR Gene Editing for T1D Cure	Preclinical stage	Potential to correct genetic predisposition to autoimmunity	Ethical and technical challenges, long-term safety unknown	[135]
	Bioartificial Pancreas (Encapsulated Insulin-Secreting Cells)	Animal models	Implantable alternative to insulin therapy	Durability and immune rejection issues	[12]
	Gut Microbiome Modulation for Autoimmune Prevention	Early human studies	May prevent autoimmune response and preserve β-cells	Needs further validation, long-term effects unknown	[136]
	Nanomedicine-Based Insulin Delivery	Preclinical stage	More efficient insulin absorption, reduced injections	No human trials yet, safety concerns	[137]

**Table 4:** The role of probiotics in T1D management.

Probiotic Type	Key Details	Type of Study (Participants)	Duration of Treatment	Key Findings	Reference
**Multispecies Probiotic (*Lactobacillus acidophilus, Lactobacillus plantarum, Bifidobacterium lactis, Saccharomyces boulardii*)**	Commonly found in fermented foods; supports gut health and immune modulation.	Randomized, double-blind, placebo-controlled clinical trial (91)	6 months	Significant reduction in HbA1c, fasting glucose, and total cholesterol	[167,168]
**Multispecies Probiotic *(Lactobacillus casei, Bifidobacterium bifidum*)**	Frequently used in probiotic supplements; aids in gut microbiome stability.	Randomized controlled trial (60)	12 weeks	Significant improvement in glycemic control and lipid profile.	[166]
***Lactobacillus rhamnosus*** **GG**	Well-researched strain for gut health; found in dairy-based probiotic products.	Randomized controlled trial (33)	6 months	No significant effect on β-cell function or HbA1c levels.	[169]
**Multispecies Probiotic (*Bifidobacterium longum, Lactobacillus bulgaricus, Streptococcus thermophilus*)**	Used in dairy products and probiotic formulations; supports digestion and metabolism.	Randomized, double-blinded, placebo-controlled pilot study (50)	12 weeks	Significant reduction in fasting blood glucose levels.	[170]
**Synbiotic Supplement (Probiotics + Prebiotics)**	Combination of beneficial bacteria and dietary fibers for gut health.	Randomized controlled trial (130)	6 months	Study protocol; results pending.	[171]

## Data Availability

No new data were created.

## Abbreviations

The following abbreviations are used in this manuscript:

T1D	Type 1 diabetes
CGM	Continuous glucose monitoring
AI	Artificial intelligence
CRISPR	Clustered Regularly Interspaced Short Palindromic Repeats
WHO	World Health Organization
IDF	International Diabetes Federation
ISPAD	International Society for Pediatric and Adolescent Diabetes
CSII	Continuous subcutaneous insulin infusion
FDA	Food and Drug Administration
IT	Information technology
MAFA	Musculoaponeurotic fibrosarcoma oncogene homolog A
PDX1	Pancreatic and duodenal homeobox 1
ARX	Aristaless-related homeobox
DKA	Diabetic ketoacidosis
SGLT2	Sodium-Glucose Co-Transporter 2
TREG	Regulatory T-cell
GDPR	General Data Protection Regulation
HIPAA	Health Insurance Portability and Accountability Act
